# EEG-based major depressive disorder recognition by neural oscillation and asymmetry

**DOI:** 10.3389/fnins.2024.1362111

**Published:** 2024-02-14

**Authors:** Xinyu Liu, Haoran Zhang, Yi Cui, Tong Zhao, Bin Wang, Xiaomeng Xie, Sixiang Liang, Sha Sha, Yuxiang Yan, Xixi Zhao, Ling Zhang

**Affiliations:** ^1^Beijing Key Laboratory of Mental Disorders, National Clinical Research Center for Mental Disorders and National Center for Mental Disorders, Beijing Anding Hospital, Capital Medical University, Beijing, China; ^2^Advanced Innovation Center for Human Brain Protection, Capital Medical University, Beijing, China; ^3^Gnosis Healthineer Co. Ltd., Beijing, China

**Keywords:** major depressive disorder, electroencephalography, neural oscillation, asymmetry, diagnostic regression model

## Abstract

**Background:**

Major Depressive Disorder (MDD) is a pervasive mental health issue with significant diagnostic challenges. Electroencephalography (EEG) offers a non-invasive window into the neural dynamics associated with MDD, yet the diagnostic efficacy is contingent upon the appropriate selection of EEG features and brain regions.

**Methods:**

In this study, resting-state EEG signals from both eyes-closed and eyes-open conditions were analyzed. We examined band power across various brain regions, assessed the asymmetry of band power between the hemispheres, and integrated these features with clinical characteristics of MDD into a diagnostic regression model.

**Results:**

Regression analysis found significant predictors of MDD to be beta2 (16–24 Hz) power in the Prefrontal Cortex (PFC) with eyes open (*B* = 20.092, *p* = 0.011), beta3 (24–40 Hz) power in the Medial Occipital Cortex (MOC) (*B* = −12.050, *p* < 0.001), and beta2 power in the Right Medial Frontal Cortex (RMFC) with eyes closed (*B* = 24.227, *p* < 0.001). Asymmetries in beta1 (12–16 Hz) power with eyes open (*B* = 28.047, *p* = 0.018), and in alpha (8–12 Hz, *B* = 9.004, *p* = 0.013) and theta (4–8 Hz, *B* = −13.582, *p* = 0.008) with eyes closed were also significant predictors.

**Conclusion:**

The study confirms the potential of multi-region EEG analysis in improving the diagnostic precision for MDD. By including both neurophysiological and clinical data, we present a more robust approach to understanding and identifying this complex disorder.

**Limitations:**

The research is limited by the sample size and the inherent variability in EEG signal interpretation. Future studies with larger cohorts and advanced analytical techniques are warranted to validate and refine these findings.

## 1 Introduction

Major Depressive Disorder (MDD) has become one of the three leading causes for years lived with disability with more than 264 million people affected worldwide (James et al., 2018). For those affected, MDD means personal suffering, reduced functioning and quality of life, social withdrawal, risk for co-morbid medical condition and increased mortality risk (Kessler and Bromet, 2013). Traditional diagnostic practices for MDD, while valuable, rely on subjective assessments that may not adequately reflect the biological foundations of the disorder (Dean and Keshavan, 2017). This highlights the pressing need for objective biomarkers that can enhance diagnostic precision and inform personalized treatment strategies (Ivanets et al., 2021; Carrle et al., 2023).

Neurophysiological methods such as electroencephalography (EEG) have gained traction as potential tools for elucidating the neural correlates of MDD (Lei et al., 2022). EEG’s high temporal resolution enables the detection of subtle changes in brain oscillations that are often associated with MDD, offering a window into the underlying neurophysiology of the disorder. The relevance of EEG in MDD diagnosis is further underscored by its potential to reveal altered spectral power within specific frequency bands linked to the disorder’s emotional dysfunctions (Cao et al., 2022; Han et al., 2022; Teng et al., 2022; Huang et al., 2023; Kavanaugh et al., 2023; Han et al., 2023a). Despite these advances, challenges persist in translating these findings into clinical practice, with issues such as inter-individual variability and the influence of medication status affecting the utility of EEG as a standalone diagnostic tool (Watts et al., 2022). To accurately identify patients with MDD, researchers have explored various analysis methods for EEG signals. Hasanzadeh et al. (2019) integrated multiple nonlinear features to achieve a classification accuracy of up to 91.3%. Bai et al. (2021) employed the k-Nearest Neighbors (KNN) model to analyze the complexity features of the gamma band, reaching an accuracy of 79.63%, while a random forest classifier achieved an accuracy of 65.94% for the fractal dimension of the beta band.

EEG data, which quantifies cortical activity through time series analysis methods like fast Fourier transformation, is invaluable in studying MDD. Recent research has confirmed the significance of EEG frequency band power (Wang et al., 2023; Han et al., 2023b), particularly noting increased alpha and theta power in patients in the early stages of depression (Grin-Yatsenko et al., 2010). This supports the notion that specific EEG patterns, such as alpha activity, play a functional role in MDD and its treatment response. Moreover, the BDNF Val66Met polymorphism has been linked to EEG alpha power, with the MetMet variant associated with low-voltage alpha EEG in MDD patients, suggesting a genetic influence on EEG characteristics (Zoon et al., 2013). Research by Yang et al. (2023) supports the effectiveness of using power spectral density (PSD) features for examining EEG signals across frontal, temporal, and central regions. Their findings suggest that these combined regions yield the highest accuracy in detecting MDD.

Further studies indicate that depression affects the brain’s hemispheres asymmetrically, leading to distinct patterns across various regions (Jiang et al., 2021). This asymmetry in brain activity is a critical aspect of how depression manifests and is detectable through EEG. Chang et al. (2011) reported the expected pattern of decreased alpha power at right frontal sites relative to the left, suggesting a hyperactive right and hypoactive left prefrontal cortex in depression. The assessment of resting-state EEG signals from different brain areas and frequency bands has been consistently highlighted as important, offering insights into the neural mechanisms behind emotional processes. Particularly significant is the practice of combining EEG data from both eyes-open and eyes-closed conditions into a single analytical model (Han et al., 2023b). This integrated approach provides a comprehensive understanding of brain activity, including its asymmetries, which is crucial for developing robust biomarkers for conditions like MDD (Ladeira et al., 2020). Despite its potential, the application of this integrative method in MDD research is still rare, emphasizing the need for further exploration in this area to enhance the diagnosis and understanding of depression.

In this study, we analyzed the resting-state EEG characteristics of individuals with MDD and healthy controls under both eyes-closed (EC) and eyes-open (EO) conditions. We focused on the neural oscillation of different brain regions and the asymmetry of band power between hemispheres. These features, along with clinical characteristics of the disease, were incorporated into a diagnostic regression model for MDD. In contrast to traditional EEG-based methods for MDD classification, which often focus on singular frequency band analysis or simple lateralization indexes, our approach represents a significant advancement. We integrate multi-regional band power assessments with hemispheric asymmetry analysis in both eyes-closed and eyes-open conditions, providing a more detailed picture of the brain’s electrical activity. This method acknowledges the dynamic nature of EEG signals and their variability with different states of arousal, which has been overlooked in previous studies. By doing so, we aim to capture the intricate neural oscillation patterns that are more indicative of MDD, potentially leading to improved diagnostic accuracy.

## 2 Method

### 2.1 Participants

The study was conducted at Beijing Anding Hospital from July 2022 to May 2023. All patients receiving psychiatric services at the hospital during this period were consecutively invited to participate in the survey. The inclusion criteria consisted of age between 18 and 65 years, a diagnosis of MDD according to the Diagnostic and Statistical Manual of Mental Disorders, 5th edition (DSM-5) and a total score of ≥17 on the 17-item Hamilton Depression Rating Scale (HDRS-17). Exclusion criteria included the presence of a severe and unstable medical or surgical condition, a history of alcohol or substance abuse/dependence, and a diagnosis of dementia or other evident cognitive impairments. Healthy controls (HCs) were recruited from the community through advertisements. The study protocol received approval from the Ethics Committee of Beijing Anding Hospital (Registration Number: 2020-106), and all participants provided written informed consent following a thorough explanation of the study details. This study has completed clinical registration on https://www.chictr.org.cn/ (Clinical Trial Registration Number: ChiCTR2200059053).

### 2.2 Data collection and measurements

The primary socio-demographic and clinical data were collected using a form designed for this study. We collected the basic demographic information of participants, including age and gender. The severity of the depressive and anxious symptoms was measured using the HDRS-17 and the Hamilton Anxiety Scale-14 (HAMA-14).

### 2.3 EEG signal acquisition and data processing

EEG signal acquisition was meticulously performed, capturing resting-state EEG data from participants through a structured protocol. Each participant underwent a sequence of resting-state recordings starting with a 10-min eyes-closed session, followed by a 30-s rest period, and concluding with a 10-min eyes-open session. The eyes-open recording was conducted with the participant seated comfortably in a chair, facing a monitor placed 100 cm away with a black background and a white fixation cross located the central line of sight. Participants were instructed to remain calm and relaxed, minimize head and limb movements, and consistently gaze at the fixation cross to reduce the impact of blinking and eye movements on the EEG signal.

Similarly, during the 10-min eyes-closed session, participants were asked to maintain a quiet, alert state. If a participant began to doze off, an auditory warning from the experimenter was issued. Any instances of warnings, opening eyes, or other non-resting states were marked and noted. Upon completion of the experiment, participants were assisted in washing off the conductive EEG paste from their scalp.

Data were obtained from 19 Ag/AgCl electrode channels using the advanced Neuracle system, which operates at a sampling rate of 1,000 Hz. While referencing the Cz electrode, we ensured that impedance was maintained below 50 kΩ, a level that our system’s high-resolution amplifiers can accommodate without compromising data integrity. To control for potential distortion and fluctuations in both noise and signal, we implemented several measures: The EEG recording environment was carefully controlled for electrical and ambient noise. Participants were prepared adequately to minimize impedance, including skin preparation to reduce resistance. The Neuracle system was calibrated before each recording session to ensure optimal signal acquisition. Continuous monitoring of impedance levels was performed throughout the recording to detect and rectify any deviations promptly. Signal quality was assessed in real-time, with any segments affected by artifacts being marked for exclusion from subsequent analyses.

#### 2.3.1 EEG preprocessing

EEG data preprocessing utilized the EEGLAB toolbox within MATLAB R2013a for bandpass filtering (1–40 Hz) and notch filtering (49–51 Hz), followed by downsampling to 500 Hz. Two-second epochs were employed for artifact rejection and further analysis. Eye movement artifacts were removed by independent component analysis.

Epochs with voltage excursions beyond ±150 μV were excluded. Subsequently, data were re-referenced to the average reference, and spectral power and asymmetry were computed for the 2-s epochs.

#### 2.3.2 Power spectrum

Power spectrum analysis was conducted using a Fast Fourier Transform (FFT) algorithm to quantify brain activity in the frequency domain (Han et al., 2021a,b), with power represented by the average instantaneous power of the analytic signal. Relative power for each frequency band was determined by normalizing the absolute power to the total broadband power, encompassing delta (1–4 Hz), theta (4–8 Hz), alpha (8–12 Hz), beta1 (12–16 Hz), beta2 (16–24 Hz), and beta3 (24–40 Hz). Electrodes were categorized into ten regions of interest (ROIs) for focused analysis: Prefrontal Cortex (PFC, including FP1, FP2, and Fz), Right Medial Frontal Cortex (RMFC, including Fz, F4, and F8), Left Medial Frontal Cortex (LMFC, including Fz, F3, and F7), Central Cortex (CC, including C3, C4, and Cz), Parietal Cortex (PP, including P3, P4, and Pz), Left Temporal Cortex (LT, including F7, T3, and T5), Right Temporal Cortex (RT, including F8, T4, and T6), Medial Occipital Cortex (MOC, including O1, Pz, and O2), Right Medial Occipital Cortex (RMOC, including P4, O2, and Pz), and Left Medial Occipital Cortex (LMOC, including P3, O1, and Pz).

### 2.4 Statistical analysis

Statistical analyses were performed with R version 4.0.3 and MATLAB 2013b. Group comparisons for demographic and clinical variables were conducted using chi-square tests and t-tests, with a significance level set at *p* < 0.05 (two-tailed). Binary logistic regression analyzed potential predictors of MDD, and the model’s performance was validated using 10-fold cross-validation to ensure robustness. The receiver operating characteristic (ROC) curve analysis determined the optimal cut-off points for neural oscillation power values between MDD patients and healthy controls, calculating the area under the curve, sensitivity, and specificity. Pearson correlation was employed to examine the relationship between neural oscillation characteristics and symptom severity.

## 3 Results

### 3.1 Demographic and clinical characteristics of MDD and HC

We collected and analyzed data for 86 MDD patients and 83 healthy controls. The male-to-female ratios and age distributions did not differ significantly between the groups (*χ*^2^ = 1.279, *p* = 0.258 for gender; *t* = −0.218, *p* = 0.827 for age). There was no significant difference in education level between the two groups (*t* = 0.44, *p* = 0.66). The distribution of married and unmarried individuals did not differ significantly between the two groups (*χ*^2^ = 0.03, *p* = 0.93). The MDD group presented with a mean HDRS-17 score of 24.14 and a mean HAMA score of 20.09 (Table 1).

**Table 1:** Demographic and clinical characteristics of MDD and HC groups.

Characteristics	MDD	HC	Statistics	
	(*n* = 86)	(*n* = 83)	χ2/t	*p* value
Sex (male/female)	31/55	37/46	1.28	0.26
Age (years)^†^	26.16 ± 6.29	26.41 ± 8.24	−0.22	0.83
Education level (years) ^†^	13.06 ± 3.15	12.84 ± 3.19	0.44	0.66
Married/Unmarried	57/28	54/28	0.03	0.93
HDRS-17^†^	24.14 ± 4.81			
HAMA^†^	20.09 ± 7.00			

^†^Mean ± SD; MDD, Major depressive disorder; HC, healthy control; HDRS-17, the 17-item Hamilton Depression Rating Scale; HAMA, the Hamilton Anxiety Scale-14.

### 3.2 Spectral power and asymmetry analysis

**Figure 1:**
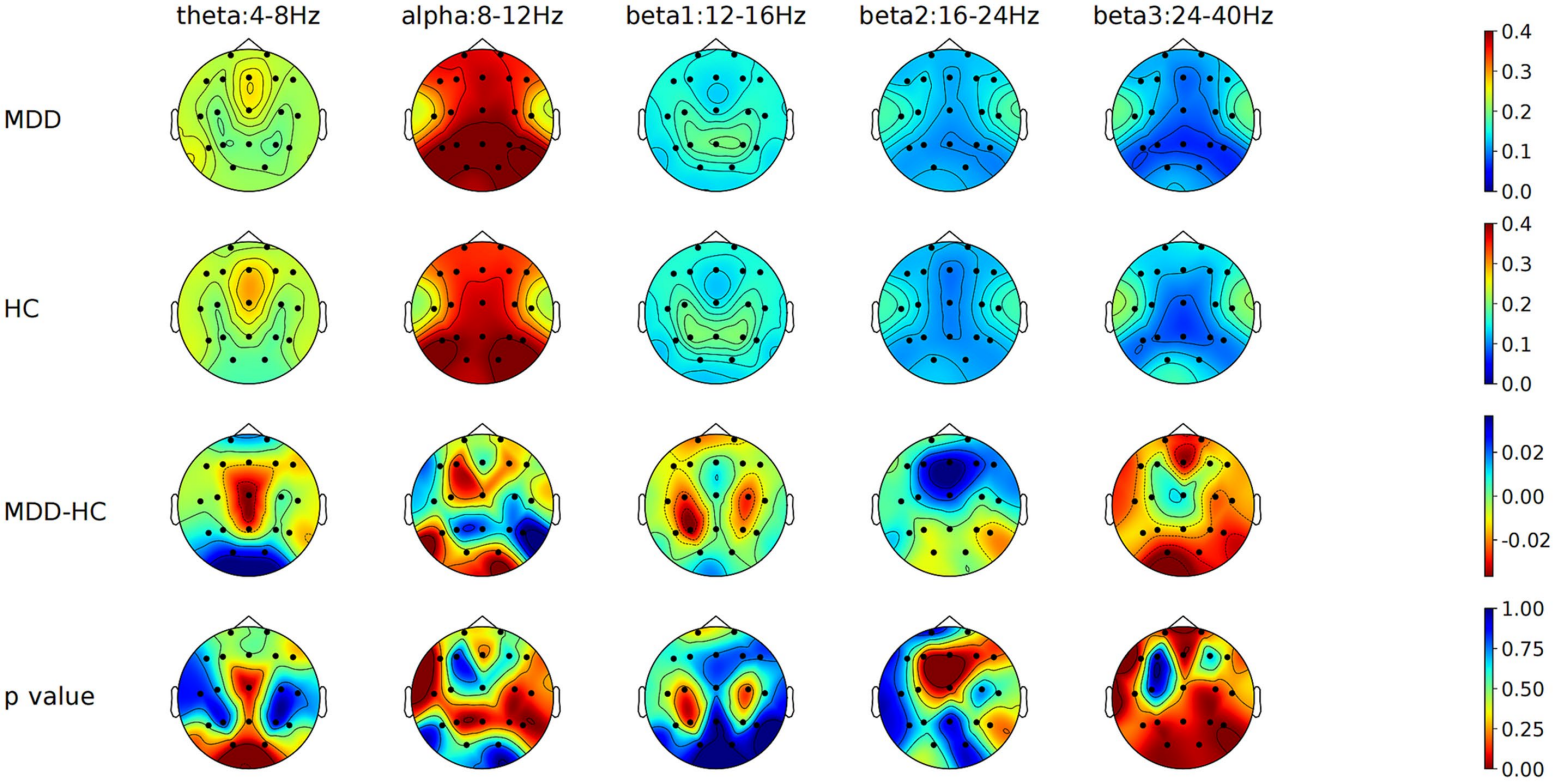
Comparison of relative power values of each frequency band in each brain region between MDD and HC groups with eyes closed. MDD, Major depressive disorder; HC, Healthy control; MDD-HC, Difference in relative power between two groups.

**Figure 2:**
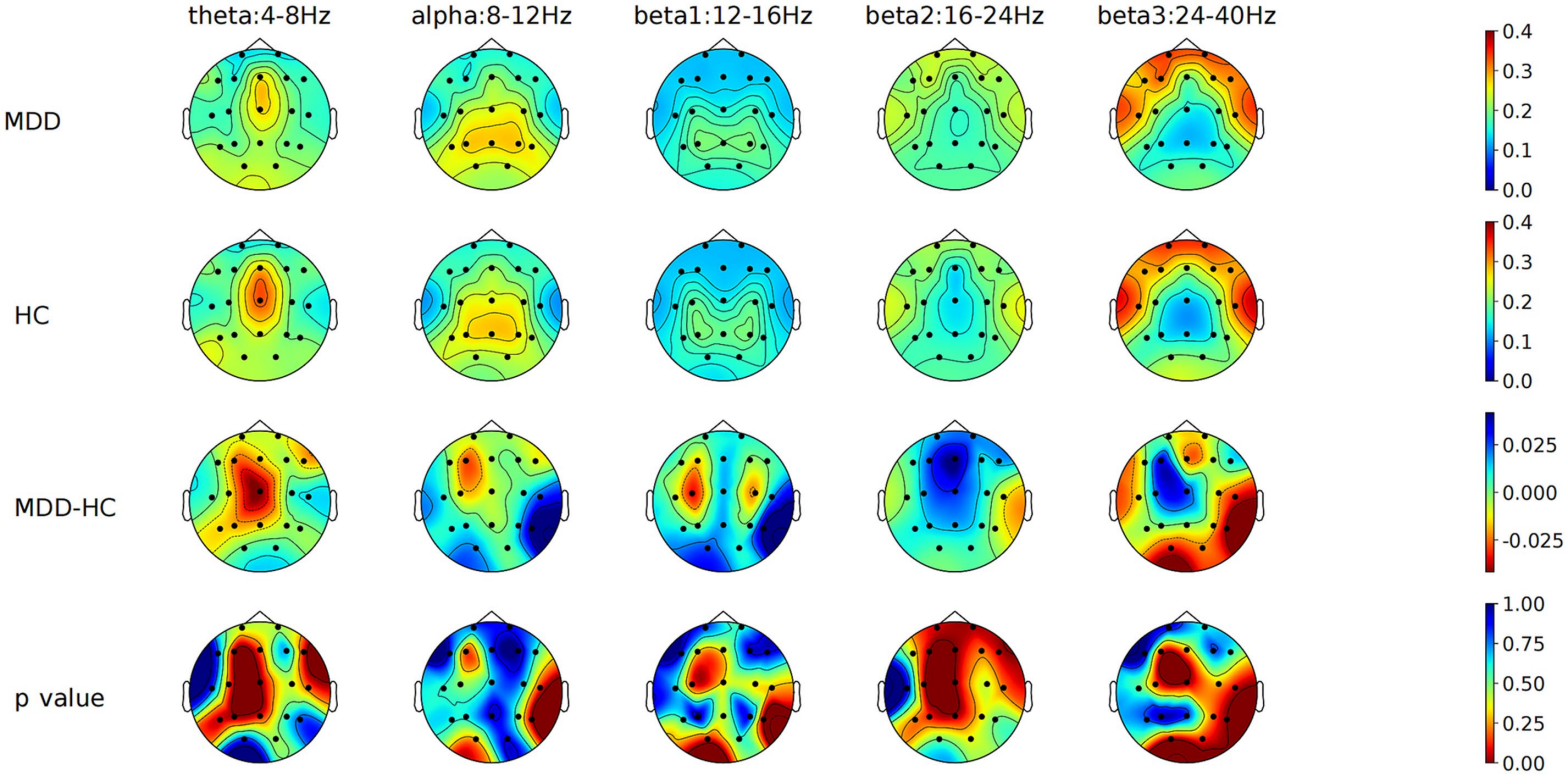
Comparison of relative power values of each frequency band in each brain region between MDD and HC groups with eyes open. MDD, Major depressive disorder; HC, Healthy control; MDD-HC, Difference in relative power between two groups.

Independent samples *t*-tests compared EEG relative band power across various brain regions between 86 MDD patients and 83 HCs. For the eyes-closed condition, MDD patients exhibited significantly higher beta2 band power in the RMFC, alpha band power in the RT, and lower beta3 band power in the RMOC compared to HCs (Figure 1; Supplementary Table S1). Under the eyes-open condition, there was a significant increase in beta2 band power in the PFC, RMFC, LMFC, and CC in MDD patients, with a notable decrease in beta3 band power in the MOC relative to HCs. After FDR correction, beta2 band power in the PFC, RMFC, and LMFC in the MDD is still significantly increased, and the trend of other parameter characteristics remains unchanged but statistically significant diminished (Figure 2; Supplementary Table S1).

Asymmetry in alpha and beta1 band power between the RT and LT regions was more pronounced in MDD patients during the eyes-open condition, while the asymmetry in beta2 and beta3 band power was significantly less marked compared to HCs. Under the eyes-closed condition, the alpha band power asymmetry between RT and LT was significantly greater in MDD patients, whereas theta band power asymmetry was significantly reduced. After FDR correction, the statistically significant asymmetric differences between the two groups in beat2 band power at RT-LT in the eyes-open condition and in theta band power in the eyes-closed condition at RT-LT were attenuated, and all other parameters remained statistically significant (Table 2).

**Table 2:** Comparison of relative power asymmetry between left and right brain regions in each frequency band in MDD and HC groups.

BR	FB	HC (EO)	MDD (EO)	Statistics (EO)			HC (EC)	MDD (EC)	Statistics (EC)		
		Mean ± SD	Mean ± SD	P1	P2	Effect Size (Cohen’s d)	Mean ± SD	Mean ± SD	P1	P2	Effect Size (Cohen’s d)
RMFC-LMFC	Theta	0.003 ± 0.043	0.005 ± 0.035	0.782	0.782	−0.043	0.003 ± 0.024	0.000 ± 0.018	0.377	0.505	0.148
	Alpha	0.002 ± 0.027	0.007 ± 0.025	0.189	0.27	−0.203	0.001 ± 0.025	0.000 ± 0.024	0.929	0.929	0.014
	Beta1	−0.001 ± 0.013	0.002 ± 0.014	0.21	0.21	−0.193	−0.002 ± 0.008	0.000 ± 0.009	0.361	0.472	−0.141
	Beta2	−0.002 ± 0.025	−0.003 ± 0.020	0.666	0.772	0.066	−0.001 ± 0.010	0.001 ± 0.013	0.353	0.353	−0.143
	Beta3	−0.003 ± 0.059	−0.012 ± 0.054	0.347	0.52	0.145	−0.001 ± 0.039	−0.002 ± 0.028	0.963	0.963	0.007
RMOC-LMOC	Theta	−0.007 ± 0.035	0.000 ± 0.023	0.141	0.423	−0.228	−0.003 ± 0.030	−0.004 ± 0.027	0.828	0.828	0.033
	Alpha	0.004 ± 0.025	0.000 ± 0.024	0.27	0.27	0.17	0.019 ± 0.043	0.009 ± 0.034	0.127	0.191	0.236
	Beta1	0.004 ± 0.014	0.001 ± 0.015	0.18	0.21	0.207	0.003 ± 0.017	0.005 ± 0.015	0.472	0.472	−0.111
	Beta2	−0.001 ± 0.014	−0.001 ± 0.012	0.772	0.772	0.045	−0.007 ± 0.017	−0.004 ± 0.014	0.227	0.34	−0.187
	Beta3	−0.001 ± 0.049	0.000 ± 0.032	0.799	0.799	−0.039	−0.013 ± 0.052	−0.006 ± 0.032	0.316	0.474	−0.155
RT-LT	Theta	−0.015 ± 0.045	−0.007 ± 0.053	0.339	0.509	−0.148	0.003 ± 0.024	0.000 ± 0.018	0.037	0.112	0.323
	Alpha	−0.010 ± 0.032	0.007 ± 0.041	0.004	0.012	−0.449	0.001 ± 0.025	0.003 ± 0.024	0.003	0.009	−0.463
	Beta1	−0.003 ± 0.018	0.008 ± 0.022	<0.001	0.001	−0.547	−0.002 ± 0.008	0.000 ± 0.009	0.283	0.472	−0.166
	Beta2	0.008 ± 0.033	−0.003 ± 0.033	0.037	0.111	0.324	−0.001 ± 0.010	0.001 ± 0.013	0.095	0.285	0.258
	Beta3	0.019 ± 0.055	−0.006 ± 0.071	0.013	0.038	0.388	−0.001 ± 0.039	−0.002 ± 0.028	0.222	0.474	0.188

MDD, Major depressive disorder; HC, healthy control; SD, standard error; EO, eyes open; EC, eyes closed; PFC, Prefrontal Cortex; RMFC, Right Medial Frontal Cortex; LMFC, Left Medial Frontal Cortex; LT, Left Temporal Cortex; RT, Right Temporal Cortex; RMOC, Right Medial Occipital Cortex; LMOC, Left Medial Occipital Cortex; P1, Uncorrected *p*-value; P2, FDR-corrected *p*-value.

### 3.3 Regression analysis of discrepant data

**Figure 3:**
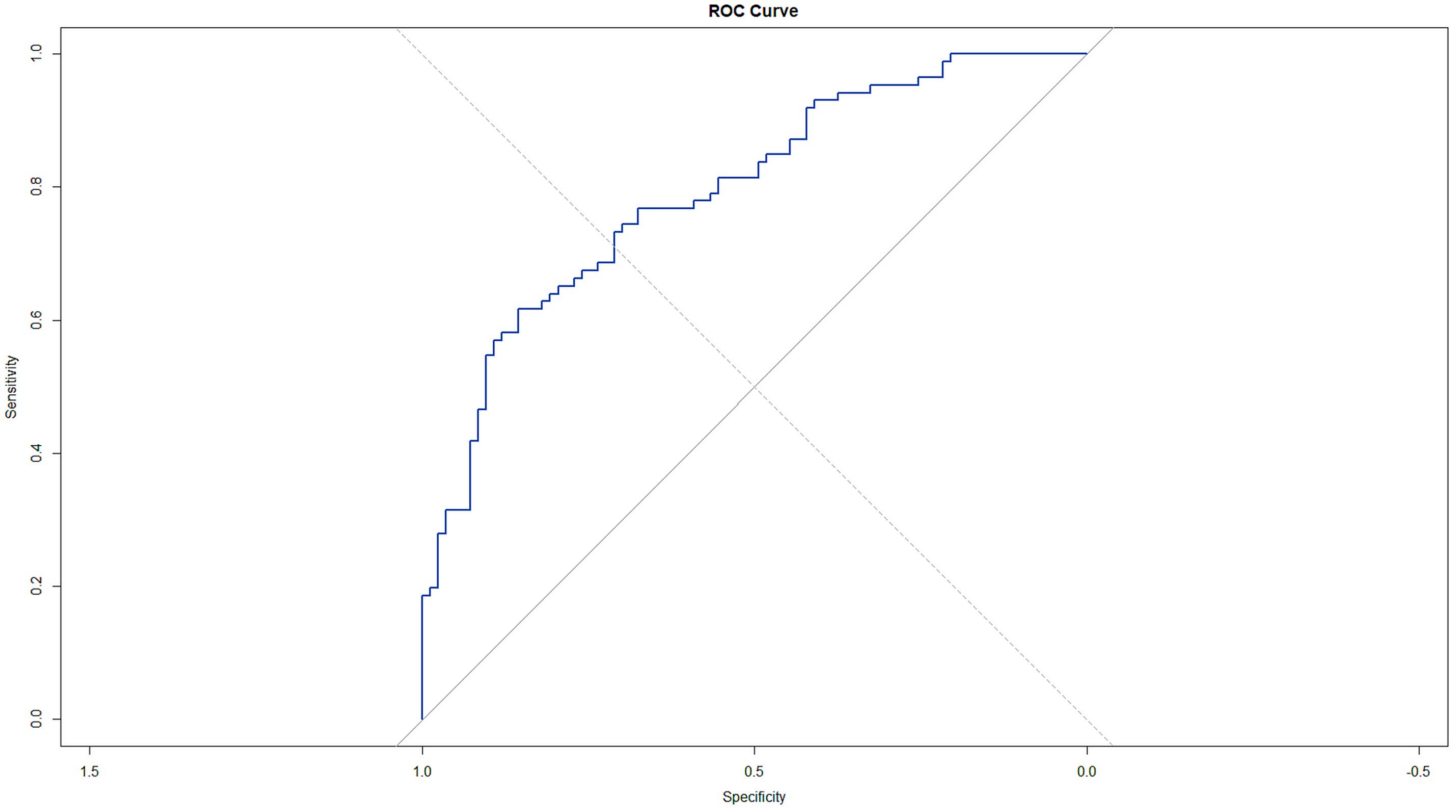
ROC curve analysis of the regression model in predicting Major depressive disorder.

Binary logistic regression was utilized in 86 MDD patients and 83 HCs to identify potential EEG predictors of MDD, with the diagnosis as the dependent variable. EEG band powers showing significant differences in t-tests (beta2 in PFC, RMFC, LMFC, CC, beta3 in MOC for eyes-open; beta2 in RMFC, alpha in RT, beta3 in RMOC, RMFC, RT, MOC, LMOC for eyes-closed) and power asymmetries (alpha, beta1, beta2, beta3 between RT and LT for eyes-open; alpha, theta between RT and LT for eyes-closed) were included as independent variables. The analysis determined that beta2 band power in PFC with eyes open (*B* = 20.092, *p* = 0.011), beta3 in MOC with eyes open (*B* = −12.050, *p* < 0.001), beta2 in RMFC with eyes closed (*B* = 24.227, *p* < 0.001), asymmetry in beta1 band power between RT and LT with eyes open (*B* = 28.047, *p* = 0.018) and in alpha (*B* = 9.004, *p* = 0.013) and theta (*B* = −13.582, *p* = 0.008) with eyes closed were significant predictors of MDD (Table 3). The model’s performance was validated using 10-fold cross-validation to ensure robustness. The average area under the ROC curve (AUC) from the cross-validation was approximately 0.7709, indicating a fair discrimination ability of the model. The sensitivity and specificity obtained were around 68.47 and 66.94%, respectively. To assess the statistical significance of the model’s predictive capability, we calculated the 95% confidence interval for the AUC, which ranged from 0.7261 to 0.8592, not encompassing the null hypothesis value of 0.5 and thus confirming that the model performed significantly better than chance. The ROC curve was plotted to visually represent the model’s performance, with a blue line indicating the trade-off between sensitivity and specificity across different thresholds, and a grey dashed line representing the performance of a random classifier (Figure 3).

**Table 3:** Binary logistic regression of potential predictors of MDD.

State			*B*	SE	*p*
Eyes open	EEG relative band power	beta2 in PFC	20.092	7.872	0.011
		beta2 in RMFC	2.630	9.583	0.784
		beta2 in LMFC	−3.890	9.560	0.684
		beta2 in CC	−1.021	6.036	0.866
		beta3 in MOC	−12.050	3.078	<0.001
	Relative power asymmetry between left and right brain regions	alpha RT-LT	12.511	11.657	0.283
		beta1 RT-LT	28.047	11.859	0.018
		beta2 RT-LT	−0.514	9.718	0.958
		beta3 RT-LT	2.887	5.556	0.603
Eyes closed	EEG relative band power	beta3 in PFC	−2.030	3.188	0.524
		beta2 in RMFC	24.227	5.941	<0.001
		alpha in RT	1.070	2.172	0.622
		beta3 in RT	−4.128	4.113	0.316
		beta3 in MOC	10.822	10.618	0.308
		beta3 in RMOC	−3.355	7.857	0.669
		beta3 in LMOC	−16.962	10.146	0.095
	Relative power asymmetry between left and right brain regions	theta RT – LT	−13.582	5.119	0.008
		alpha RT – LT	9.004	3.640	0.013

PFC, Prefrontal Cortex; RMFC, Right Medial Frontal Cortex; LMFC, Left Medial Frontal Cortex; CC, Central Cortex; LT, Left Temporal Cortex; RT, Right Temporal Cortex; MOC, Medial Occipital Cortex; RMOC, Right Medial Occipital Cortex; LMOC, Left Medial Occipital Cortex.

### 3.4 ROC curve analysis

ROC curve analysis in 86 MDD patients and 83 HCs evaluated the EEG band powers’ predictive capabilities for MDD. The AUC indicated that beta2 band power in PFC with eyes open (AUC = 0.655, *p* < 0.001) could predict MDD with a sensitivity of 0.744 and specificity of 0.544, with an optimal cut-off point of 0.298 according to the Youden index. Similarly, the AUC for the asymmetry in beta1 band power between RT and LT with eyes open (AUC = 0.650, *p* < 0.001) predicted MDD with a sensitivity of 0.465 and specificity of 0.692, with an optimal cut-off of 0.308. For eyes-closed conditions, alpha (AUC = 0.590, *p* = 0.002) and theta (AUC = 0.639, *p* = 0.04) asymmetries between RT and LT were also predictive of MDD, with respective sensitivities of 0.279 and 0.523 and specificities of 0.892 and 0.723 at optimal cut-offs of 0.022 and 0.246.

### 3.5 Correlation analysis in MDD

**Figure 4:**
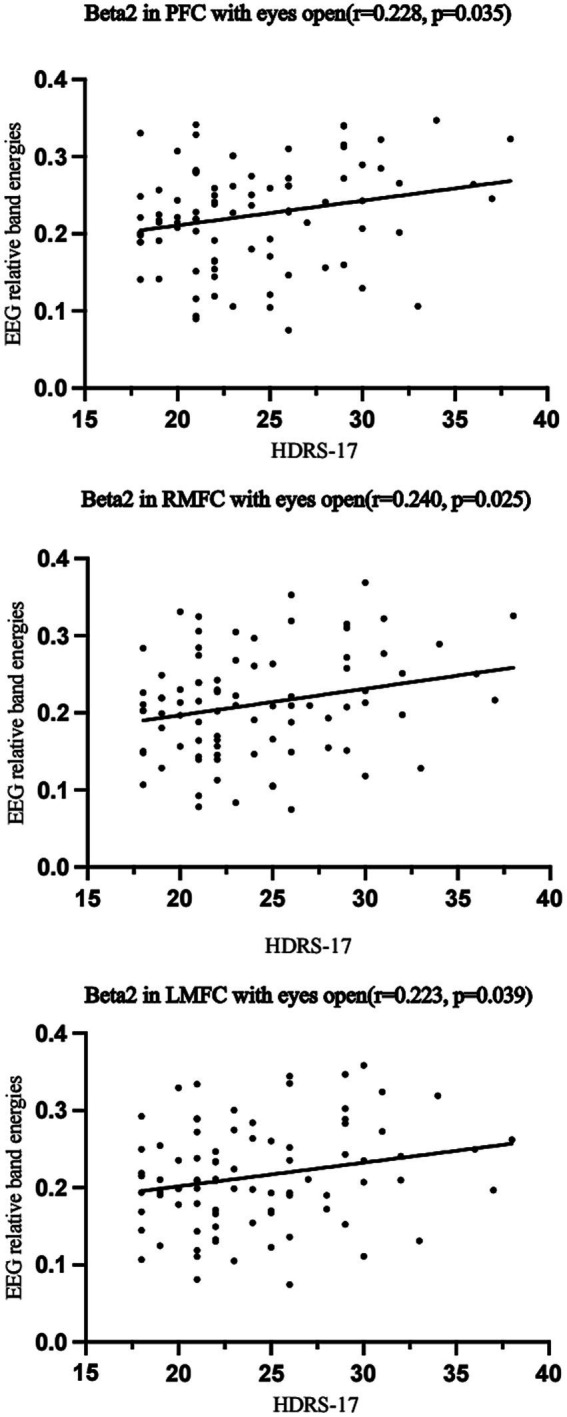
Pearson correlation analysis of EEG band power and asymmetry with HDRS-17 scores in patients with Major depressive disorder. MDD, Major depressive disorder; PFC, Prefrontal Cortex; RMFC, Right Medial Frontal Cortex; LMFC, Left Medial Frontal Cortex.

Pearson correlation analysis explored the relationship between clinically relevant indicators and EEG band power in 86 MDD patients. Significant positive correlations emerged between HDRS-17 scores and beta2 band power in PFC (*r* = 0.228, *p* = 0.035), RMFC (*r* = 0.240, *p* = 0.025), and LMFC (*r* = 0.223, *p* = 0.039) with eyes open (Figure 4).

## 4 Discussion

In the present study, we investigated the alterations in EEG spectral power in individuals with MDD and explored their potential as objective markers for diagnosis and personalized treatment. Our findings revealed significant changes in beta2 activity in the prefrontal cortex, and alterations in beta3 power in the occipital cortex, alpha and beta power in the parietal and temporal regions. Binary logistic regression identified significant EEG predictors of MDD, including beta2 power in PFC with eyes open, beta3 power in MOC with eyes open, beta2 power in RMFC with eyes closed, asymmetry in beta1 power between RT and LT with eyes open, and asymmetry in alpha and theta power with eyes closed. The model’s performance indicating fair discrimination ability. The ROC curve visually represented the model’s performance, demonstrating its superiority over a random classifier. The regression model involving multiple variables demonstrates a better predictive ability for depression than any individual factor. Furthermore, the correlation between EEG spectral features and clinical indicators suggests the potential of EEG as a monitoring tool for the clinical course of MDD (Baskaran et al., 2012; Olbrich and Arns, 2013).

Spectral analysis revealed that individuals with MDD exhibited a marked elevation in beta2 relative band power in the PFC, RMFC, LMFC, and CC when their eyes were open. This augmentation in beta2 band power aligns with neurocognitive models of MDD, which suggest hyperactivity in specific brain circuits, correlating with rumination and negative cognitive biases—hallmarks of depression (Siegle et al., 2007). Such increased beta activity may mirror frontal lobe dysregulation, supporting the frontal lobe hypothesis of depression that associates changes in frontal brain activity with depressive symptoms (Pizzagalli, 2011; Sharpley et al., 2023b). Furthermore, Claverie et al. (2016) provide compelling evidence for the prognostic value of EEG spectral features, particularly beta2 main peak frequency, in identifying vulnerability to depression. This research, conducted on a rat model, demonstrates that individuals exhibiting lower beta2 main peak frequency prior to exposure to stressors were more likely to become vulnerable to depression, as indicated by persistent low serum BDNF levels. The persistence of altered EEG patterns in vulnerable animals across different time points—before stress exposure, immediately after, and one month later—suggests that these electrophysiological markers are stable indicators of susceptibility to depression.

Conversely, in the occipital region, particularly the MOC, the MDD group displayed a significant reduction in beta3 power, potentially indicating anomalies in visual processing or the occipital lobe’s role in mood regulation (Bruder et al., 2001; Liu et al., 2022). Our study’s findings of altered band power in the parietal regions are corroborated by literature that implicates the parietal lobe in the neural circuitry of depression (Fales et al., 2008; Liu et al., 2023). The right parietal cortex, in particular, is associated with attentional control and emotional regulation—capabilities often compromised in MDD (Fales et al., 2008). Additionally, the parietal cortex’s contribution to the default mode network (DMN), known to be disrupted in MDD, further supports our observations (Greicius et al., 2007; Sheline et al., 2009). The DMN is linked to self-referential mental activity, frequently exhibiting a negative bias in depression (Hamilton et al., 2012), which could manifest as altered EEG spectral power in the parietal regions.

Consistent reports of alpha band power alterations in the parietal regions suggest cortical hypoactivation in MDD (Pollock and Schneider, 1990; Bruder et al., 2001). Simultaneously, some studies propose that increased alpha power in the right parietal cortex reflects an internal attentional focus (Benedek et al., 2014) and a causal relationship between parietal alpha activity and spatial auditory attention (Deng et al., 2019). Our findings, consistent with these studies, propose that parietal hypoactivation may serve as a stable neurophysiological marker for MDD. Under the eyes-closed condition, our study identified an increase in alpha power in the RT region and alterations in beta3 power in the RMOC and LMOC among participants with MDD. These results are consistent with existing literature, which reports an increase in alpha band power during eyes-closed rest, potentially more pronounced in MDD (Thatcher, 2012; Chandler et al., 2022). The RT region, known for its role in emotional processing and memory—areas often compromised in MDD—may account for the specific regional increase in alpha power (Drevets, 1998; Bruder et al., 2001).

The power asymmetry between the LT and RT regions in the alpha and beta bands underscores the potential lateralization of brain activity in MDD (Messerotti Benvenuti et al., 2019). The beta3 power asymmetry between the RMOC and LMOC could reflect the lateralized dysfunction in MDD, which is believed to disrupt affective processing and attention (Bruder et al., 2001). Given the occipital cortex’s primary role in visual processing, changes in beta3 power may indicate broader sensory processing issues in MDD (Kaiser et al., 2015). Moreover, these occipital lobe changes in beta3 power might relate to disruptions in the DMN, which includes occipital components and is affected in MDD (Greicius et al., 2007; Sheline et al., 2009). The DMN is associated with self-referential thought and mind-wandering, often negatively biased in MDD, which could be reflected in altered EEG patterns (Mayberg, 1997; Siegle et al., 2007). The observed imbalances may indicate disrupted interhemispheric communication, a factor implicated in the pathophysiology of depression (Uhlhaas and Singer, 2010). This lateralization has been noted in EEG studies, where alpha-band asymmetries correlated with emotional processing and depression severity (Bruder et al., 2001; Xie et al., 2023).

Furthermore, alterations in interhemispheric alpha power have been linked to functional disconnection between the cerebral hemispheres, potentially underlying the cognitive and affective disturbances in MDD (McVoy et al., 2019). Beta-band activity in the LT region is associated with language and executive functions, which are often impaired in MDD, suggesting that beta-band imbalances may correspond to the observed difficulties in cognitive control and verbal communication (Siegle et al., 2007; Pizzagalli, 2011). Additionally, research indicates that the LT region is involved in approach-related emotional processing, while the RT is associated with withdrawal-related emotions (Harmon-Jones et al., 2010). The alpha and beta power imbalance between these regions could reflect the emotional dysregulation and anhedonia commonly reported in MDD. Neuroimaging studies have supported the presence of structural and functional abnormalities in the temporal lobes of individuals with MDD, reinforcing the concept of lateralized dysfunction (Drevets, 1998; Lai, 2014). These abnormalities may be connected to disrupted connectivity within the limbic–cortical networks, which is essential for emotional regulation and stress response (Mayberg, 1997; Sheline et al., 2009).

In our results, binary logistic regression analyses of EEG data have identified certain spectral power features as robust predictors of MDD. Notably, these features include the relative power of the beta2 band in the PFC with eyes open and asymmetries in the power of the beta1, alpha, and theta bands between the RT and LT regions, with eyes open and closed, respectively. The PFC is known for its role in executive functions and emotion regulation, both of which are often impaired in MDD (Siegle et al., 2007; Hiser and Koenigs, 2018). Beta2 activity, in particular, has been associated with active cognitive processes and attention (Engels et al., 2010), and its dysregulation may reflect the cognitive disturbances observed in individuals with MDD (Clark et al., 2017). When eyes are open, asymmetry in beta1 power between the RT and LT regions could indicate the lateralized processing of emotional stimuli and stress response, which are frequently disrupted in MDD. Asymmetries in alpha power, particularly with eyes closed, have been linked to altered arousal and vigilance states, which are characteristic features of MDD (Sharpley et al., 2023a). Furthermore, theta power is associated with memory and emotional processing, and its alteration may correspond to the memory deficits and negative bias in emotional processing characteristic of MDD (H. Jiang et al., 2022). These electrophysiological markers offer a window into the underlying neural mechanisms of MDD and may enhance the accuracy of diagnostic procedures when combined with traditional clinical evaluations (Pizzagalli, 2011; Jaworska and Protzner, 2013). By providing a quantitative measure of brain activity, EEG can offer a more nuanced understanding of the disorder (Baskaran et al., 2012; Olbrich and Brunovsky, 2021).

The analysis of the ROC curve for the predictive utility of single EEG spectral power in MDD resulted in modest Area Under the Curve (AUC) values, such as 0.655 for beta2 in the PFC with eyes open, indicating a fair level of discriminative ability. However, when multiple EEG spectral power and asymmetry were included in a binary logistic regression model, the average AUC improved to 0.7709, indicating a more robust discrimination ability. This variability highlights the complexity of MDD as a disorder and the challenges in identifying a single biomarker with high diagnostic accuracy (Insel et al., 2010). While the modest AUC values obtained from ROC curve analyses of EEG features in predicting MDD do not diminish the potential value of these measures, they emphasize the importance of a multimodal diagnostic approach that integrates EEG, clinical assessments, and other biomarkers. Previous studies have also indicated that composite EEG measurement indices, such as the Antidepressant Treatment Response Index (ATR), exhibit strong predictive accuracy in determining treatment response in MDD. For instance, retrospective analysis of an initial study involving subjects with MDD treated with selective serotonin reuptake inhibitors (SSRIs) or venlafaxine demonstrated that ATR predicted response with an accuracy of 70%, with 82% sensitivity and 54% specificity (Iosifescu et al., 2009). Furthermore, in the Biomarkers for Rapid Identification of Treatment Effectiveness in Major Depression (BRITE-MD) study, ATR showed predictive value by achieving an accuracy of 74% in predicting response and remission, with a sensitivity of 58%, specificity of 91%, positive predictive accuracy of 88%, and negative predictive accuracy of 67% (Leuchter et al., 2009). Our approach aligns with contemporary psychiatric practice, which emphasizes the integration of biological data to inform diagnosis and treatment response, ultimately aims to improve outcomes for individuals with MDD (Insel et al., 2010; Markiewcz, 2017).

Correlation analysis of EEG relative band power with clinical indicators in MDD patients has provided insightful data, revealing a significant positive relationship between HDRS-17 scores and increased beta2 activity in various frontal regions, including the PFC, with coefficients ranging from *r* = 0.223 to *r* = 0.240 (Koller-Schlaud et al., 2020). This suggests that as the severity of depression increases, so does the beta2 activity in these areas. The positive correlation between HDRS-17 scores and increased beta2 activity in frontal regions suggests that EEG spectral features could serve as objective indicators for monitoring the clinical course of MDD.

However, this study is not without limitations. Given the relatively small sample size of our study, the sensitivity and specificity reported here may not accurately reflect what would be obtained in a larger, more heterogeneous population. Additionally, the cross-sectional nature of the study design precludes the ability to infer causality or the directionality of the observed relationships. Future research should include longitudinal designs to assess the temporal stability of EEG markers and their predictive value for treatment outcomes. While our findings provide valuable insights into EEG markers for MDD, they should be considered preliminary and warrant validation in larger-scale studies that can offer more definitive evidence of their generalizability. Moreover, the heterogeneity of MDD symptoms and the presence of comorbidities, such as anxiety disorders, may confound the EEG signals. Therefore, subsequent studies should consider stratifying participants based on symptom clusters or comorbid conditions (Frodl and O’Keane, 2013).

## 5 Conclusion

The study confirms the potential of multi-region EEG analysis in improving the diagnostic precision for MDD. MDD patients showed increased beta2 band power in the PFC, RMFC, LMFC, and CC under eyes-open conditions, and increased beta2 in RMFC and alpha in RT under eyes-closed conditions. Conversely, beta3 band power was lower in MDD across multiple regions. Notably, asymmetries in alpha and beta bands between right and left temporal cortices emerged as strong predictors of MDD. These EEG markers, along with clinical scores, provide potent diagnostic indicators for MDD.

## Data availability statement

The raw data supporting the conclusions of this article will be made available by the authors, without undue reservation.

## Ethics statement

The studies involving humans were approved by the Ethics Committee of Beijing Anding Hospital. The studies were conducted in accordance with the local legislation and institutional requirements. The participants provided their written informed consent to participate in this study.

## Author contributions

XL: Writing – original draft, Writing – review & editing, Methodology, Supervision, Validation. HZ: Writing – original draft, Writing – review & editing, Data curation, Formal analysis, Investigation, Methodology, Software, Supervision, Validation. YC: Methodology, Software, Visualization, Writing – review & editing, Project administration, Supervision, Validation. TZ: Methodology, Software, Visualization, Writing – review & editing, Supervision, Validation. BW: Data curation, Software, Visualization, Writing – review & editing, Project administration, Resources. XX: Data curation, Methodology, Visualization, Writing – review & editing. SL: Data curation, Software, Validation, Visualization, Writing – review & editing. SS: Project administration, Resources, Validation, Visualization, Writing – review & editing. YY: Project administration, Resources, Writing – review & editing. XZ: Conceptualization, Data curation, Formal analysis, Funding acquisition, Investigation, Methodology, Project administration, Resources, Supervision, Writing – review & editing. LZ: Conceptualization, Formal analysis, Investigation, Methodology, Project administration, Resources, Supervision, Writing – review & editing.
