# Supplementary material for: EEG-based major depressive disorder recognition by neural oscillation and asymmetry
Source: Front Neurosci. 2024 Feb 14;18:1362111. doi: 10.3389/fnins.2024.1362111 (PMC10899403; doi:10.3389/fnins.2024.1362111)
Supplement: Supplementary file 1 [file Table_1.DOCX]

Supplementary Material Table 1. Comparison of relative power values of each frequency band in each brain region between MDD and HC groups

| BR | FB | HC(EO) | MDD (EO) | Statistics (EO) | | | HC (EC) | MDD (EC) | Statistics (EC) | | |
| --- | --- | --- | --- | --- | --- | --- | --- | --- | --- | --- | --- |
|  |  | Mean±SD | Mean±SD | P1 | P2 | Effect Size (Cohen's d) | Mean±SD | Mean±SD | P1 | P2 | Effect Size (Cohen's d) |
| PFC | theta | 0.192±0.097  0.171±0.111  0.121±0.049  0.188±0.064  0.318±0.163  0.215±0.083  0.184±0.099  0.126±0.041  0.184±0.059  0.280±0.137  0.212±0.084  0.182±0.099  0.128±0.040  0.185±0.058  0.283±0.138  0.235±0.074  0.240±0.090  0.170±0.054  0.166±0.047  0.179±0.087  0.219±0.071  0.273±0.110  0.196±0.057  0.159±0.051  0.143±0.079  0.188±0.076  0.190±0.096  0.149±0.039  0.204±0.057  0.261±0.114  0.172±0.069  0.180±0.094  0.146±0.041  0.212±0.057  0.280±0.115  0.219±0.076  0.242±0.103  0.170±0.044  0.169±0.048  0.191±0.102  0.214±0.071  0.257±0.107  0.183±0.050  0.165±0.049  0.172±0.093  0.221±0.073  0.252±0.104  0.178±0.048  0.165±0.048  0.173±0.094 | 0.174±0.104  0.164±0.107  0.125±0.040  0.224±0.067  0.303±0.145  0.200±0.098  0.181±0.105  0.129±0.035  0.211±0.068  0.268±0.128  0.195±0.098  0.173±0.102  0.128±0.034  0.215±0.066  0.279±0.130  0.214±0.076  0.235±0.102  0.165±0.044  0.184±0.057  0.191±0.088  0.203±0.075  0.279±0.109  0.198±0.050  0.173±0.061  0.137±0.065  0.178±0.086  0.194±0.108  0.147±0.036  0.211±0.066  0.261±0.123  0.171±0.075  0.200±0.103  0.154±0.041  0.208±0.060  0.256±0.110  0.224±0.080  0.250±0.101  0.177±0.041  0.175±0.053  0.163±0.075  0.215±0.077  0.264±0.105  0.187±0.045  0.173±0.056  0.151±0.070  0.215±0.077  0.264±0.104  0.186±0.045  0.175±0.057  0.150±0.071 | 0.270 | 0.593 | 0.170 | 0.246±0.081 0.344±0.140 0.147±0.050 0.111±0.042 0.138±0.101 0.256±0.086 0.339±0.134 0.144±0.047 0.113±0.040 0.135±0.094 0.252±0.084 0.339±0.137 0.145±0.047 0.114±0.041 0.136±0.092 0.233±0.087 0.348±0.127 0.166±0.053 0.122±0.042 0.117±0.084 0.198±0.092 0.396±0.127 0.199±0.061 0.110±0.044 0.085±0.063 0.219±0.078 0.351±0.133 0.157±0.048 0.129±0.048 0.132±0.090 0.216±0.079 0.344±0.137 0.159±0.047 0.132±0.050 0.137±0.087 0.185±0.081 0.398±0.156 0.164±0.055 0.116±0.048 0.125±0.099 0.186±0.087 0.410±0.144 0.179±0.057 0.109±0.045 0.102±0.080 0.189±0.080 0.391±0.143 0.176±0.056 0.116±0.047 0.116±0.090 | 0.246±0.121  0.365±0.148  0.145±0.065  0.123±0.059  0.110±0.070  0.243±0.115  0.359±0.142  0.144±0.061  0.130±0.060  0.113±0.069  0.242±0.115  0.358±0.141  0.145±0.061  0.129±0.058  0.115±0.067  0.219±0.102  0.367±0.127  0.160±0.059  0.131±0.054  0.111±0.063  0.195±0.111  0.43±0.139  0.190±0.080  0.105±0.047  0.068±0.048  0.226±0.105  0.367±0.134  0.151±0.062  0.133±0.063  0.115±0.080  0.209±0.105  0.387±0.141  0.157±0.066  0.128±0.055  0.109±0.070  0.202±0.111  0.418±0.151  0.167±0.072  0.112±0.053  0.093±0.077  0.196±0.114  0.431±0.148  0.179±0.077  0.107±0.050  0.078±0.061  0.200±0.106  0.422±0.143  0.174±0.074  0.111±0.051  0.084±0.067 | 0.980 | 0.980 | 0.004 |
|  | alpha |  |  | 0.675 | 0.839 | 0.065 |  |  | 0.338 | 0.442 | -0.148 |
|  | beta1 |  |  | 0.522 | 0.865 | -0.099 |  |  | 0.791 | 0.998 | 0.041 |
|  | beta2 |  |  | 0.001 | 0.005 | -0.544 |  |  | 0.109 | 0.363 | -0.248 |
|  | beta3 |  |  | 0.517 | 0.697 | 0.100 |  |  | 0.033 | 0.067 | 0.330 |
| RMFC | theta |  |  | 0.296 | 0.593 | 0.161 |  |  | 0.403 | 0.819 | 0.129 |
|  | alpha |  |  | 0.839 | 0.839 | 0.031 |  |  | 0.353 | 0.442 | -0.143 |
|  | beta1 |  |  | 0.606 | 0.865 | -0.080 |  |  | 0.923 | 0.998 | -0.015 |
|  | beta2 |  |  | 0.006 | 0.020 | -0.428 |  |  | 0.037 | 0.290 | -0.324 |
|  | beta3 |  |  | 0.557 | 0.697 | 0.090 |  |  | 0.087 | 0.116 | 0.265 |
| LMFC | theta |  |  | 0.224 | 0.593 | 0.188 |  |  | 0.535 | 0.819 | 0.096 |
|  | alpha |  |  | 0.567 | 0.839 | 0.088 |  |  | 0.360 | 0.442 | -0.141 |
|  | beta1 |  |  | 0.947 | 0.947 | -0.010 |  |  | 0.929 | 0.998 | 0.014 |
|  | beta2 |  |  | 0.003 | 0.014 | -0.468 |  |  | 0.058 | 0.290 | -0.294 |
|  | beta3 |  |  | 0.843 | 0.936 | 0.031 |  |  | 0.093 | 0.116 | 0.260 |
| CC | theta |  |  | 0.062 | 0.593 | 0.289 |  |  | 0.340 | 0.819 | 0.147 |
|  | alpha |  |  | 0.759 | 0.839 | 0.047 |  |  | 0.312 | 0.442 | -0.156 |
|  | beta1 |  |  | 0.455 | 0.865 | 0.115 |  |  | 0.460 | 0.998 | 0.114 |
|  | beta2 |  |  | 0.024 | 0.060 | -0.350 |  |  | 0.251 | 0.627 | -0.177 |
|  | beta3 |  |  | 0.365 | 0.697 | -0.140 |  |  | 0.603 | 0.603 | 0.080 |
| PP | theta |  |  | 0.174 | 0.593 | 0.210 |  |  | 0.848 | 0.942 | 0.030 |
|  | alpha |  |  | 0.724 | 0.839 | -0.055 |  |  | 0.081 | 0.405 | -0.270 |
|  | beta1 |  |  | 0.796 | 0.884 | -0.040 |  |  | 0.394 | 0.998 | 0.132 |
|  | beta2 |  |  | 0.124 | 0.247 | -0.238 |  |  | 0.560 | 0.722 | 0.090 |
|  | beta3 |  |  | 0.549 | 0.697 | 0.092 |  |  | 0.054 | 0.091 | 0.298 |
| LT | theta |  |  | 0.443 | 0.738 | 0.118 |  |  | 0.655 | 0.819 | -0.069 |
|  | alpha |  |  | 0.813 | 0.839 | -0.037 |  |  | 0.442 | 0.442 | -0.118 |
|  | beta1 |  |  | 0.697 | 0.872 | 0.060 |  |  | 0.492 | 0.998 | 0.106 |
|  | beta2 |  |  | 0.443 | 0.492 | -0.118 |  |  | 0.650 | 0.722 | -0.070 |
|  | beta3 |  |  | 0.982 | 0.982 | 0.003 |  |  | 0.187 | 0.207 | 0.204 |
| RT | theta |  |  | 0.878 | 0.936 | 0.024 |  |  | 0.608 | 0.819 | 0.079 |
|  | alpha |  |  | 0.196 | 0.839 | -0.200 |  |  | 0.045 | 0.405 | -0.310 |
|  | beta1 |  |  | 0.178 | 0.865 | -0.208 |  |  | 0.820 | 0.998 | 0.035 |
|  | beta2 |  |  | 0.717 | 0.717 | 0.056 |  |  | 0.650 | 0.722 | 0.070 |
|  | beta3 |  |  | 0.160 | 0.399 | 0.217 |  |  | 0.022 | 0.066 | 0.357 |
| MOC | theta |  |  | 0.673 | 0.841 | -0.065 |  |  | 0.253 | 0.819 | -0.177 |
|  | alpha |  |  | 0.600 | 0.839 | -0.081 |  |  | 0.410 | 0.442 | -0.127 |
|  | beta1 |  |  | 0.299 | 0.865 | -0.160 |  |  | 0.748 | 0.998 | -0.050 |
|  | beta2 |  |  | 0.390 | 0.487 | -0.133 |  |  | 0.628 | 0.722 | 0.075 |
|  | beta3 |  |  | 0.046 | 0.315 | 0.310 |  |  | 0.019 | 0.066 | 0.365 |
| RMOC | theta |  |  | 0.936 | 0.936 | -0.012 |  |  | 0.545 | 0.819 | -0.093 |
|  | alpha |  |  | 0.676 | 0.839 | -0.064 |  |  | 0.344 | 0.442 | -0.146 |
|  | beta1 |  |  | 0.568 | 0.865 | -0.088 |  |  | 0.998 | 0.998 | 0.000 |
|  | beta2 |  |  | 0.285 | 0.407 | -0.165 |  |  | 0.768 | 0.768 | 0.045 |
|  | beta3 |  |  | 0.095 | 0.315 | 0.259 |  |  | 0.026 | 0.066 | 0.345 |
| LMOC | theta |  |  | 0.584 | 0.835 | 0.084 |  |  | 0.468 | 0.819 | -0.112 |
|  | alpha |  |  | 0.488 | 0.839 | -0.107 |  |  | 0.161 | 0.442 | -0.217 |
|  | beta1 |  |  | 0.300 | 0.865 | -0.160 |  |  | 0.877 | 0.998 | 0.024 |
|  | beta2 |  |  | 0.250 | 0.407 | -0.178 |  |  | 0.451 | 0.722 | 0.116 |
|  | beta3 |  |  | 0.071 | 0.315 | 0.279 |  |  | 0.011 | 0.066 | 0.395 |

Note: MDD: Major depressive disorder; HC: healthy control; SD: standard error; EO: eyes open; EC: eyes closed; PFC: Prefrontal Cortex; RMFC: Right Medial Frontal Cortex; LMFC: Left Medial Frontal Cortex; LT: Left Temporal Cortex; RT: Right Temporal Cortex; RMOC: Right Medial Occipital Cortex; LMOC: Left Medial Occipital Cortex; P1: Uncorrected p-value; P2: FDR-corrected P-value.
